# The mechanism of dynamic switching between M1/M2 phenotypes of microglia in neuropathic pain: a narrative review

**DOI:** 10.3389/fnmol.2026.1834555

**Published:** 2026-05-26

**Authors:** Yunyan Mao, Longsheng Xu, Ming Yao

**Affiliations:** 1 College of Life Sciences and Medicine, Zhejiang Sci-Tech University, Hangzhou, Zhejiang, China; 2 Department of Anesthesia and Pain Medicine, Affiliated Hospital of Jiaxing University, Jiaxing, China

**Keywords:** microglia, neuroinflammation, neuropathic pain, polarization, targeted therapy

## Abstract

Neuropathic pain (NeP) is a chronic pain disorder caused by damage to the somatosensory system, often presenting with long-lasting symptoms and limited efficacy of standard pharmacological treatments. Recent evidence indicates that the microglial activation exists on a dynamic and environment-dependent continuum and this plasticity critically influences the development and maintenance of neuropathic pain. During the polarization process, microglia can release different mediators, and these functional states dynamically regulate neuroinflammation and central sensitization, thereby exerting a profound impact on the occurrence and persistence of NeP. Among these phenotypes, dynamic functional transitions play a critical role in shaping pain outcomes. By releasing pro-inflammatory cytokines such as TNF-α and IL-1β, pain is intensified, whereas anti-inflammatory signaling contributes to protective and reparative effects. This review highlights the critical molecular mechanisms underlying microglial differentiation, including the P2X7, TLR4/NF-κB, and USP19/FOXO1 signaling pathways. It also discusses how these pathways influence the regulation of pain circuits. We also discuss intervention strategies targeting polarization balance, including the potential and challenges of emerging therapeutic approaches such as small-molecule inhibitors, biologics, natural products, and nano-delivery systems. Upcoming studies need to look beyond the M1/M2 dichotomy and explore the complexity of microglial behavior, decode the phenotypic spectrum of microglia at different stages of pain by means of multi-omics technologies, and pay attention to gender differences and translational bottlenecks, so as to promote the development of novel precision analgesic strategies based on neuroimmune regulation.

## 1 Introduction

Pain represents a major global public health burden, severely impairing patients’ quality of life and often accompanied by comorbidities such as anxiety and depression (Tozaki-Saitoh and Tsuda, 2019). Neuropathic pain (NeP), a subtype of chronic pain, is directly caused by damage to or disease of the somatosensory nervous system, accounting for approximately 7 to 10% of patients with chronic pain. Its typical characteristics such as burning sensations and electric shock-like pain cause severe suffering to patients (Xu et al., 2021). Despite the widespread use of existing medications (e.g., gabapentinoids, tricyclic antidepressants), most patients achieve only partial relief and experience significant adverse effects, which highlights the unmet therapeutic needs for NeP (Parusel et al., 2023). This has led researchers to explore its complex underlying mechanisms in hopes of finding new treatment targets.

The development of NeP involves peripheral sensitization and central sensitization, in which the neuroimmune crosstalk in the spinal dorsal horn plays a particularly crucial role (Tsuda et al., 2023; Zhang et al., 2024b). Microglial polarization is increasingly recognized as a key contributor to neuropathic pain, with growing evidence suggesting that targeting its phenotypic dynamics may offer a promising therapeutic approach (Chen et al., 2018; Gui et al., 2020; Long et al., 2025; Zou et al., 2025). Microglia exhibit a dynamic polarization process, transitioning between anti-inflammatory and pro-inflammatory phenotypes. Microglia with the pro-inflammatory phenotype secrete proinflammatory cytokines and neurotoxic substances, while those with the anti-inflammatory phenotype exert neuroprotective effects by promoting tissue repair and regeneration. Following neuropathic pain, microglia rapidly respond to abnormal microenvironmental signals and shift toward pro-inflammatory or anti-inflammatory phenotypes, which exerts a critical impact on the prognosis. Studies have shown that (Deng et al., 2025) converting microglia from the proinflammatory phenotype to the anti-inflammatory phenotype can effectively alleviate the symptoms of neuropathic pain. As the intrinsic immune sentinels of the central nervous system, microglia play a central role in this process (Kwok et al., 2021). Upon nerve injury or inflammatory stimulation, resting microglia are rapidly activated, undergoing profound morphological and functional changes (Banati et al., 2004; Nimmerjahn et al., 2005). Activated microglia do not exist in a single uniform state. They polarize into distinct phenotypes in response to signals from their microenvironment. Among these, the proinflammatory M1 and the anti-inflammatory M2 subtypes have been studied most extensively (Long et al., 2024). These two phenotypes possess distinctly different molecular markers and functional characteristics. M1 microglia release large amounts of proinflammatory cytokines (e.g., TNF-α, IL-1β, and IL-6), along with neurotoxic substances. These factors can increase neuronal excitability and contribute to pain development. Conversely, M2 microglia secrete anti-inflammatory cytokines (e.g., IL-10 and TGF-β), as well as neurotrophic factors, which promote tissue repair and limit inflammatory damage (Lively and Schlichter, 2018; Wang et al., 2019; Nan et al., 2024). Emerging research indicates that the balance of this polarization state directly influences the progression of NeP, and the transition from acute to chronic pain is closely linked to sustained pro-inflammatory activation of microglia (Inoue and Tsuda, 2018).

As a result, mapping the regulatory network of microglial polarization and identifying strategies to shift the balance from pro-inflammatory toward anti-inflammatory/repair-associated phenotypes have become major research priorities in the mechanistic study of NeP and the development of novel analgesics. This review provides a comprehensive overview of the role of microglial activation state in NeP, summarizes critical molecular pathways, and discusses potential therapeutic interventions targeting this process.

## 2 Methods

This review was conducted through a structured search of the published literature to summarize current knowledge on the role of microglial phenotypic shift in neuropathic pain and to discuss potential therapeutic strategies targeting this process. Electronic databases including PubMed, Web of Science, and Scopus were searched for relevant articles published up to December 2025 (see Supplementary Table S1), yielding a total of 1,559 records (PubMed: *n* = 647; Web of Science: *n* = 491; Scopus: *n* = 421). After removal of duplicates (*n* = 445), 1,114 records remained for title and abstract screening (Supplementary Figure S1). The search strategy combined keywords related to microglial biology and neuropathic pain, including “microglia,” “microglial polarization,” “M1,” “M2,” “neuropathic pain,” “neuroinflammation,” “P2X7,” “TLR4/NF-κB,” and “microglia-targeted therapy.” Inclusion and exclusion criteria are presented in a flow diagram (see Supplementary Table S2).

During the initial screening, 865 records were excluded due to irrelevance to microglia (*n* = 303), pain (*n* = 288), or lack of polarization-related data (*n* = 274). The remaining 249 articles were assessed in full text for eligibility. Of these, 142 studies were further excluded for the following reasons: absence of original polarization data (n = 48), non-English publications (*n* = 12), lack of relevant signaling pathways (*n* = 35), focus on other glial cell types (*n* = 47). After the above screening process, a total of 107 studies relevant to this work were identified and included for citation.

The collected literature was then qualitatively analyzed and organized into thematic sections, focusing on key molecular pathways, regulatory networks, and emerging therapeutic strategies associated with microglial functional sataes in neuropathic pain. The aim of this approach was to highlight potential directions for future research.

## 3 Neuropathic pain: concepts, classification and clinical challenges

Neuropathic pain (NeP) is defined by the International Association for the Study of Pain (IASP) as “pain arising as a direct consequence of a lesion or disease affecting the somatosensory nervous system” (Ochoa, 2009; Xu et al., 2021). Unlike nociceptive pain, NeP stems from direct injury to or dysfunction of the nervous system, often presenting as abnormal sensations such as burning, electric shock, pricking or pressure, and is prone to chronicity (Jayathilake et al., 2025).

Clinically, NeP has diverse classification and can be divided from an anatomical perspective into central NeP (e.g., post-stroke pain, post-spinal cord injury pain) and peripheral NeP (e.g., diabetic peripheral neuropathy, post-herpetic neuralgia), with the latter being more prevalent in clinical practice (Zhang et al., 2024b; Jayathilake et al., 2025).

NeP not only impairs patients’quality of life but also poses significant challenges in clinical management (Xu et al., 2021; Parusel et al., 2023). First-line medications (e.g., gabapentin, pregabalin, tricyclic antidepressants) are widely used, yet they are commonly associated with incomplete efficacy (pain relief of more than 50% is achieved in only about 30–40% of patients) and a high incidence of adverse reactions (Parusel et al., 2023). For gabapentinoids, somnolence and dizziness occur in 15–25% of patients, often leading to dose reduction or discontinuation. Tricyclic antidepressants are associated with anticholinergic effects (dry mouth, constipation, urinary retention) and cardiac conduction abnormalities, particularly in older adults (Crunkhorn, 2019). This highlights the urgency of moving beyond the traditional neuron-centric view, thoroughly elucidating the molecular mechanisms of NeP from novel perspectives such as neuroimmune crosstalk, and advancing the development of therapies targeting new molecular targets.

## 4 Microglia: from resting sentinels to polarized phenotypes

### 4.1 Origin, resting state and activation of microglia

**Figure 1:**
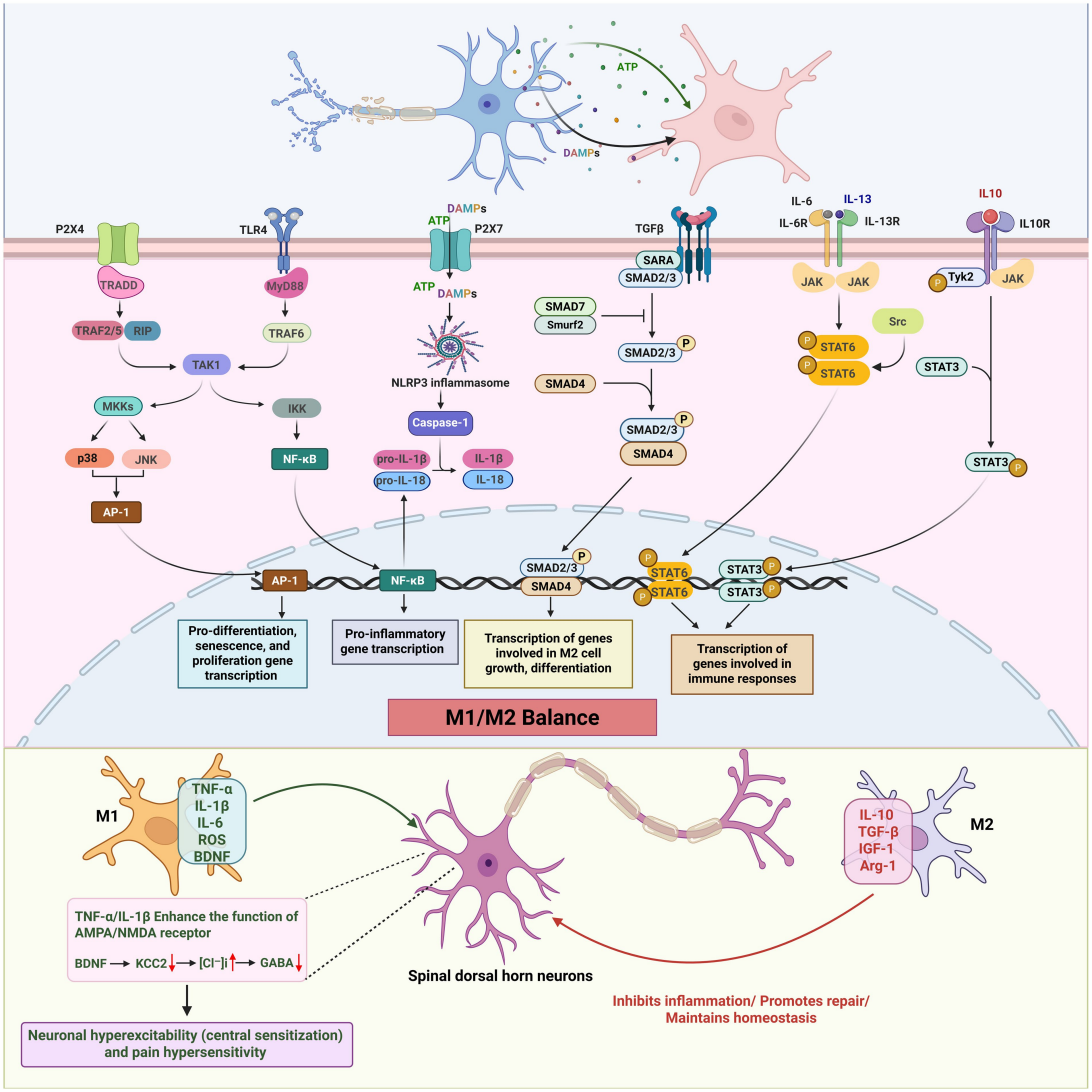
Schematic diagram of the regulatory network of spinal microglial polarization after nerve injury and its role in pain. This figure summarizes the core regulatory network of spinal microglial polarization following peripheral nerve injury and its effects on the pain circuit. Nerve injury triggers the release of signals such as ATP and DAMPs, which activate P2X4/7, TLR4, and other receptors on the microglial membrane. Through signaling axes including NF-κB and NLRP3, this activation drives microglial polarization toward the pro-inflammatory M1 phenotype, leading to the release of mediators such as TNF-α, IL-1β, and BDNF. These mediators enhance the function of neuronal AMPA/NMDA receptors and disrupt inhibitory synapses, ultimately resulting in central sensitization and hyperalgesia. Meanwhile, protective signaling pathways such as IL-10 and TGF-β/SMAD can promote the anti-inflammatory and reparative M2 phenotype, which secretes factors including IL-10 and TGF-β to counteract inflammation and facilitate the restoration of homeostasis. The dynamic balance between M1 and M2 phenotypes exerts a regulatory effect on the progression of neuropathic pain. Created with bioGDP.com.

Microglia are resident macrophages that populate the central nervous system (CNS). Derived from yolk sac progenitors, they play a central role in CNS development, homeostasis maintenance and immune surveillance (Kwok et al., 2021; Xie et al., 2021). Under physiological conditions, microglia exist in a highly dynamic “resting” state, constantly extending and retracting their processes to monitor the microenvironment (Nimmerjahn et al., 2005). Once PAMPs or DAMPs (e.g., ATP and high-mobility group box 1) are detected, microglia are rapidly activated, and the morphology of activated microglia changes from a quiescent ramified form to an activated amoeboid form (Zhao et al., 2021; Nheu et al., 2022). They perform functions such as post-injury synaptic pruning, phagocytosis of apoptotic and damaged cells, inflammatory response and tissue repair (Zhang et al., 2020; Au and Ma, 2022). This activation is characterized by an enlarged cell body, shortened processes, and migration to the injury site (Banati et al., 2004; Sivaprakasam et al., 2016) (Figure 1).

### 4.2 Polarization: moving beyond the M1/M2 dichotomy to a broader phenotypic spectrum

Emerging evidence indicates that microglia exhibit remarkable plasticity across a range of conditions (Crunkhorn, 2019), including aging, central nervous system disorders, neurodegenerative diseases, and psychiatric disorders such as depression and anxiety, indicating that targeted regulation of the M1/M2 phenotypic balance-such as inhibiting M1 proinflammatory responses or promoting M2 anti-inflammatory repair- represents an effective therapeutic strategy for CNS disorders (Li et al., 2024; Wu et al., 2024). Its functional phenotypes (i.e., polarization) are precisely regulated by local microenvironmental signals. The classical theory classifies them into two types: M1 (classical activation) and M2 (alternative activation). The M1 phenotype is mainly induced by lipopolysaccharide (LPS), interferon-γ (IFN-γ) and other factors, highly expresses surface markers (e.g., CD86 and CD16/32), and releases a large amount of proinflammatory mediators including tumor necrosis factor-α (TNF-α), interleukin-1β (IL-1β) and inducible nitric oxide synthase (iNOS), thereby exacerbating neuroinflammation and neural damage. The M2 phenotype is induced by interleukin-4 (IL-4), IL-13 and other factors (Lan et al., 2017; Jurga et al., 2020), and is characterized by high expression of CD206 and Arg-1, and secretes IL-10, transforming growth factor-β (TGF-β) and neurotrophic factors, thereby exerting anti-inflammatory effects and playing roles in promoting tissue repair and protecting neurons (Jurga et al., 2020).

Microglial phenotypic shift is increasingly recognized as a dynamic and context-dependent process rather than a binary M1/M2 classification. As highlighted by Paolicelli et al. (2022), microglia exhibit a continuum of heterogeneous states shaped by local environmental cues, temporal dynamics, and disease contexts. This refined framework suggests that microglial phenotypes are fluid and overlapping, enabling them to adaptively coordinate diverse functions, including neuroinflammation, synaptic remodeling, and tissue repair. Recent work, such as analyses using single-cell sequencing (Paolicelli et al., 2022), indicates that the phenotypes of microglia/macrophages *in vivo* form a continuous and dynamic spectrum, which is far more complex than a simple dichotomy. Sideris-Lampretsas and Malcangio (2021) highlight that microglia in pain conditions are not a homogeneous population but instead display substantial heterogeneity across spatial, temporal, and pathological contexts. In neuropathic pain, microglia adopt distinct transcriptional and functional states in response to injury-induced signals, forming diverse subpopulations with specialized roles. These subsets differentially regulate neuroinflammatory responses, synaptic remodeling, and neuronal excitability, thereby contributing to the initiation and maintenance of pain. This evidence underscores that microglial involvement in pain is highly context-dependent and cannot be adequately explained by simplified activation models. Under pathological conditions such as NeP, there may exist multiple functionally specialized subpopulations, For instance, “disease-associated microglia (DAM)” represent a unique subpopulation with distinct transcriptional signatures.

Therefore, understanding microglial responses in NeP requires a more refined perspective of the phenotypic spectrum, while the M1/M2 framework still provides a useful conceptual tool for describing the balance between their proinflammatory and reparative functions.

## 5 Core roles and dynamic regulatory networks of microglial polarization in neuropathic pain

The spinal dorsal horn, particularly laminae I and II, is a key site where neuroimmune interactions drive central sensitization. Following peripheral nerve injury, spinal microglia exhibit region-specific responses that may influence vulnerability to chronic pain and are intimately linked to their polarization state. Rather than responding uniformly, distinct microglial populations appear to participate in localized circuit remodeling. After nerve injury, dorsal horn microglia have been observed to selectively engulf synaptic contacts from both peripheral afferents and intrinsic spinal neurons. This activity can disrupt the balance between excitatory and inhibitory signaling. Such changes may contribute to altered spinal processing of sensory inputs (Yousefpour et al., 2023). For instance, the selective phagocytosis of synaptic structures and the degradation of perineuronal nets (PNNs) by dorsal horn microglia have been associated with a pro-inflammatory (M1-like) phenotype, specialized extracellular matrix structures located in lamina I that contributing to disinhibition of nociceptive circuits (Zhang et al., 2022). Conversely, the resolution of these changes in the chronic phase may depend on the emergence of repair-associated (M2-like) features. These region- and state-dependent activities underscore the importance of understanding microglial phenotypic shif dynamics in pain pathophysiology (Berta et al., 2016; Ho et al., 2020).

This synaptic pruning activity is temporally correlated with the distribution of microglial deposits on dorsal horn synapses and corresponds to the loss pattern of specific types of synapses (Yousefpour et al., 2025). In the chronic phase of neuropathic pain, activation of spinal microglia can persist for more than 3 months and may become partially independent of classical proinflammatory cytokine and chemokine signaling (Echeverry et al., 2017) (Figure 1).

### 5.1 Key signaling axes associated with a pro-inflammatory (M1-like) state

#### 5.1.1 Purinergic receptor P2X4/P2X7 axis

This axis is supported by multiple mechanistic observations. Following peripheral nerve injury, large amounts of ATP are released from damaged cells, activating both the P2X4 and P2X7 receptors on microglia (Zhao et al., 2017). The purinergic P2X7 receptor is specifically upregulated in spinal microglia after nerve injury and mediates amplification of neuroinflammatory signaling in response to extracellular ATP. Functionally, P2X7 receptor activation is closely linked to the maturation and secretion of pro-inflammatory cytokines such as IL-1β and IL-18 through the assembly of the NLRP3 inflammasome. These processes represent a central mechanism driving the chronification of neuropathic pain (Donnelly et al., 2020). Clinical evidence suggests that botulinum toxin type A (BTX-A) may alleviate neuropathic pain, at least in part, through modulation of P2X7 receptor expression (Li et al., 2016; Gui et al., 2020). Consistent with these molecular changes, treated animals exhibited sustained attenuation of pain hypersensitivity (Long et al., 2024). In contrast, activation of P2X4 receptors promotes the release of brain-derived neurotrophic factor (BDNF) via calcium-dependent signaling pathways. Although the downstream effects of P2X4 and P2X7 differ, they converge at the level of spinal nociceptive processing: P2X7-mediated cytokine release amplifies neuroinflammation, whereas P2X4-dependent BDNF signaling enhances neuronal excitability and facilitates central sensitization. In addition, potassium efflux triggered by P2X7 activation may influence membrane properties in a way that favors P2X4 receptor responsiveness, although this possibility remains to be clarified. Thus, the term “synergy” in this context reflects complementary functional contributions rather than a direct molecular interaction between the two receptors (Sorge et al., 2015; Inoue and Tsuda, 2018). Engagement of TrkB receptors on dorsal horn neurons by BDNF has been associated with reduced KCC2 expression and consequent alterations in chloride equilibrium. Such shifts in ionic homeostasis diminish inhibitory efficacy and favor increased neuronal responsiveness, a mechanism thought to underlie the development of hyperalgesia (Bethea and Fischer, 2021). Overall, P2X4 and P2X7 exhibit functional convergence in amplifying neuroimmune signaling, acting through distinct but complementary effector pathways.

#### 5.1.2 The canonical TLR4/MyD88/NF-*κ*B pathway

Toll-like receptor 4 (TLR4) plays an essential role in microglial activation. Its downstream MyD88/nuclear factor-κB (NF-κB) signaling pathway promotes polarization toward the proinflammatory M1 phenotype. Together, this signaling cascade constitutes a core mechanism driving pro-inflammatory microglial activation. Damage-released DAMPs (e.g., HMGB1, heat shock proteins) activate the downstream IKK complex via TLR4 and its adaptor protein MyD88, leading to the degradation of the NF-κB inhibitor (IκB) and the subsequent nuclear translocation of the NF-κB (p65/p50) dimer. Persistent activation of this pathway induces transcription of inflammatory mediators, including TNF-α, IL-1β, IL-6, COX-2, and iNOS, thereby stabilizing the pro-inflammatory phenotype of microglia (Anilkumar and Wright-Jin, 2024; Zhang et al., 2024a; Jiang et al., 2025). This pathway does not operate in isolation but interacts with stress-responsive kinases, including p38 MAPK and JNK, thereby strengthening inflammatory signaling cascades. Experimental studies suggest that selective inhibition of this pathway (e.g., using TAK-242 or Bt354) can rebalance microglial state modulation and alleviate hyperalgesia (Liu T. et al., 2024; Cheng et al., 2025). Binding of the κ-opioid receptor (KOR) appears to counteract pro-inflammatory microglial polarization, an effect linked to reduced activity of the TLR4/NF-κB axis. Such observations point to a regulatory interplay in which this signaling pathway can be modulated in opposing directions (Liu et al., 2020; Blaszczak et al., 2024).

#### 5.1.3 Other important regulators and interactive networks

Accumulating preclinical evidence indicates that activation of the BK channel (KCNMB3) and inhibition of the Kir6.1/K-ATP channel in microglia of the spinal dorsal horn both promote the M1 phenotype and exacerbate pain (Li et al., 2016; Imari et al., 2019). Following peripheral nerve injury, components of the cGAS–STING pathway become increasingly active within spinal microglia, where they are associated with a shift toward a pro-inflammatory phenotype. Activation of STING recruits TBK1, resulting in phosphorylation of IRF3 and induction of type I interferon signaling. In parallel, cross-regulation with NF-κB pathways amplifies downstream cytokine expression (Silveira Prudente et al., 2024; Chen et al., 2025). The chemokine receptor CX3CR1 functions as an essential molecular interface in neuron–microglia communication (Chen et al., 2025). Its ligand, CX3CL1 (fractalkine), is primarily expressed as a membrane-bound protein on neurons but can be proteolytically cleaved after nerve injury to generate a soluble form. This soluble CX3CL1 subsequently engages microglial CX3CR1 and contributes to activation of downstream inflammatory signaling pathways.

TREM2 (Triggering Receptor Expressed on Myeloid Cells 2) contributes to the control of microglial activation states. It is involved in coordinating phagocytic activity, lipid handling, and modulation of inflammatory responses, thereby influencing the balance between protective and excessive immune activation (Matteoli, 2024; Vijayan et al., 2025).

### 5.2 Protective signaling pathways associated with an anti-inflammatory/repair-associated (M2-like) state

It is vital to distinguish between the suppression of pro-inflammatory signaling and bona fide phenotypic reprogramming toward a repair-associated state. The pathways discussed below primarily exert anti-inflammatory effects and may induce expression of certain M2-associated markers, but this does not necessarily indicate commitment to a discrete M2 phenotype.

#### 5.2.1 Regulatory mechanisms governing IL-10 expression

The anti-inflammatory cytokine IL-10 is recognized as a major mediator to suppress inflammatory signaling pathways, and its expression is tightly controlled at multiple levels. Exposure to low-dose LPS has been reported to induce the deubiquitinase USP19 through mechanisms related to immune tolerance or preconditioning. It is important to note that IL-10 exerts potent anti-inflammatory effects that may promote the expression of repair-associated genes. Although IL-10 signaling can support the expression of certain M2-associated markers (e.g., Arg-1) under specific conditions, the presence of IL-10 alone should not be equated with commitment to a discrete M2 phenotype (Murray et al., 2014). By stabilizing the transcription factor FOXO1, USP19 facilitates IL-10 gene transcription. Once released, IL-10 engages IL-10R1 in either an autocrine or paracrine manner, leading to activation of the JAK1/STAT3 signaling cascade. This pathway supports the expression of genes involved in the anti-inflammatory process, including Arg-1 and Ym1, while suppressing pro-inflammatory transcriptional programs (Tao et al., 2023).

#### 5.2.2 TGF-β/SMAD pathway

Growth differentiation factor 11 (GDF11) and related ligands signal through the TGF-β receptor type I (TGF-βRI), leading to phosphorylation of receptor-regulated SMAD proteins, including SMAD2 and SMAD3. Once phosphorylated, these R-SMADs associate with the common mediator SMAD4 and translocate to the nucleus. Within the nucleus, the SMAD complex promotes transcription of anti-inflammatory genes, while simultaneously interfering with pro-inflammatory transcription factors such as NF-κB, limiting pro-inflammatory gene expression. Through these coordinated actions, TGF-β/SMAD signaling is conducive to the secretion of anti-inflammatory factors and the inhibition of the expression of pro-inflammatory factors (Liu and Zhang, 2025).

#### 5.2.3 Role of nuclear receptor NR4A2

NR4A2, an immediate early gene-derived nuclear receptor, responds rapidly to multiple stimuli. It constrains TLR4/NF-κB activity by interacting with the TLR4 promoter and by reducing TRAF6 ubiquitination. Through this regulatory influence, microglial activation state is skewed toward the suppression of the pro-inflammatory program and promotion of gene expression related to repair, accompanied by attenuation of neuroinflammatory responses (Cherry et al., 2014; Hu et al., 2025).

#### 5.2.4 Regulation of metabolic reprogramming

The promotion of repair-associated gene expression may depend on a metabolic shift away from aerobic glycolysis, as anti-inflammatory phenotypes tend to rely more on oxidative phosphorylation and fatty acid oxidation (Cherry et al., 2014), since neuroprotective genes tend to rely more on oxidative phosphorylation and fatty acid oxidation. Modulation of key metabolic enzymes (e.g., PKM2 and PFKFB3), as well as metabolic regulators including AMPK and mTOR, can alter polarization dynamics.

AMPK, a key energy sensor, generally promotes oxidative phosphorylation and fatty acid oxidation, thereby favoring a repair-associated microglial state. Activation of AMPK has been shown to suppress LPS-induced pro-inflammatory cytokine production and to enhance anti-inflammatory marker expression. Conversely, mTOR, particularly mTORC1, is a central regulator of anabolic metabolism and glycolysis. Hyperactivation of mTOR signaling promotes aerobic glycolysis and supports the pro-inflammatory phenotype. Pharmacological inhibition of mTOR (e.g., with rapamycin) has been reported to reduce inflammatory responses (Orihuela et al., 2016; Saxton and Sabatini, 2017). The balance between AMPK and mTOR activities therefore serves as a metabolic checkpoint that influences microglial functional polarization (O’Neill et al., 2016). These metabolic pathways therefore represent potential targets for therapeutic intervention (Pålsson-McDermott and O’Neill, 2020).

### 5.3 Microglial polarization imbalance in pain circuit modulation

Microglial state transitions is widely recognized as a key mechanism underlying the regulation of pain circuitry and central sensitization. Proinflammatory cytokines (e.g., TNF-α and IL-1β) released from M1-polarized microglia directly influence neurons in the spinal dorsal horn. Engagement of TNFR1 and IL-1R activates MyD88-dependent signaling and downstream kinases such as IRAK, TRAF6, and TAK1, leading to NF-κB and MAPK (p38/ERK) activation (Liu et al., 2013). These cascades promote phosphorylation of AMPA receptor subunits (e.g., GluA1 at Ser831/Ser845) and facilitate their trafficking to the postsynaptic membrane via increased interaction with scaffolding proteins like PSD-95. In parallel, IL-1β enhances NMDA receptor function through Src family kinase-mediated phosphorylation of NR2B subunits, increasing channel open probability and Ca^2+^ influx. Concurrently, TNF-α drives endocytosis of GABA receptors by disrupting gephyrin clustering, weakening inhibitory synaptic transmission. Together, these coordinated changes shift the excitatory–inhibitory balance toward hyperexcitability within spinal nociceptive circuits. The combined effect of these changes contributes to the initiation and persistence of central sensitization (Bethea and Fischer, 2021). Compared with M1-polarized microglia, M2-type cells counteract pro-inflammatory signaling through the release of IL-10, TGF-β, and related mediators. They also produce neurotrophic factors, including IGF-1 and BDNF, which support synaptic stability and facilitate tissue repair, thereby helping to re-establish neural homeostasis (Nan et al., 2024). After nerve injury, the persistence of pain reflects a changing balance between different signaling networks.

In conclusion, microglia dynamically transition between M1 and M2 phenotypes to finely tune the neuroinflammatory environment and synaptic plasticity. Through this process, they exert bidirectional control over the initiation, maintenance, and resolution of pain. This integrated “polarization-signaling-circuit” framework provides an important theoretical basis for developing targeted analgesic strategies aimed at microglia.

### 5.4 Underexplored dimension: sex differences

Particularly, significant sexual dimorphism exists in the activation and polarization of microglia. Studies have demonstrated that the intensity of spinal microglial responses and associated signaling pathways (e.g., P2X4) differ between male and female animals following nerve injury (Berta et al., 2016). Sex-dependent variations in pain signaling have been increasingly recognized. In male models, microglial activation is often a dominant contributor to pain hypersensitivity, but in females, adaptive immune components-particularly T cells-may exert stronger influence. Hormonal factors such as estradiol and testosterone, together with sex chromosome-associated genes including TLR7, are thought to shape these distinctions and may underlie observed differences in neuropathic pain prevalence and treatment response.

Interleukin-35 (IL-35), a member of the IL-12 cytokine family, has recently emerged as an important immunoregulatory factor in neuropathic pain (Zhu and Shan, 2020). It is mainly produced by regulatory T cells and exerts potent anti-inflammatory effects. Existing studies have shown that IL-35 can effectively alleviate neuropathic pain behaviors by suppressing neuroinflammation and modulating microglial activation (Cuitavi et al., 2023). In particular, IL-35 has been reported to inhibit the production of pro-inflammatory cytokines and promote the expression of anti-inflammatory mediators, thereby contributing to a shift in microglial functional state. However, it remains unclear whether this effect represents full phenotypic reprogramming or a partial suppression of pro-inflammatory signaling. Interestingly, accumulating evidence suggests that (Fiore et al., 2024) the analgesic effects of IL-35 exhibit significant sex differences. In experimental models, IL-35-mediated attenuation of pain hypersensitivity appears to be more pronounced in male animals, whereas its efficacy in females is relatively limited. One possible explanation for this sexually dimorphic effect lies in the fundamental differences in neuroimmune signaling pathways between males and females. Previous studies have demonstrated that microglia play a dominant role in mediating neuropathic pain in male rodents, whereas in females, adaptive immune cells, particularly T lymphocytes, contribute more prominently to pain processing (Jiang et al., 2020). Given that IL-35 primarily exerts its anti-inflammatory effects through modulation of microglial activity and regulation of cytokine production, its therapeutic efficacy may therefore be more pronounced in males, in whom microglia represent the primary effector cells in pain hypersensitivity. In addition, sex hormones such as estrogen and testosterone may further influence microglial responsiveness to IL-35 signaling, potentially by modulating intracellular pathways or receptor expression (Crespo-Castrillo and Arevalo, 2020). Sex chromosome-linked factors may also contribute to differential immune regulation, thereby amplifying the observed dimorphism.

Collectively, these findings suggest that the sex-specific effects of IL-35 are likely attributable to differences in cellular targets and neuroimmune regulatory mechanisms rather than a uniform pharmacological response. This highlights the importance of considering sex as a critical biological variable in the development of microglia-targeted therapies for neuropathic pain.

### 5.5 Context-dependent activation of polarization pathways

Some networks promote M1 microglial polarization, while others support M2-associated regulatory programs. This dynamic interplay occurs within the local microenvironment. Microglial activation represents a common feature across neuropathic pain (NeP) of diverse etiologies, including chemotherapy-induced peripheral neuropathy (CIPN), diabetic neuropathy, traumatic nerve injury, and nerve compression. Despite this shared response, the phenotypic and functional profiles of microglia exhibit notable differences depending on the underlying cause. For instance, in traumatic nerve injury and nerve compression models, microglia are rapidly activated in the spinal dorsal horn and contribute to central sensitization through pathways such as P2X4/P2X7 and TLR4 signaling (Inoue and Tsuda, 2018). In contrast, diabetic neuropathy is characterized by a more chronic, low-grade inflammatory state, with sustained microglial activation driven by metabolic dysfunction and oxidative stress (Chong and Souayah, 2025). Chemotherapy-induced neuropathy involves distinct microglial responses associated with mitochondrial dysfunction and altered immune signaling, which may differ from classical injury-induced activation patterns (Starobova and Vetter, 2017). To sum up, while microglial activation is a convergent mechanism underlying NeP, its temporal dynamics, signaling pathways, and functional outcomes vary substantially across etiologies, highlighting the need for etiology-specific therapeutic strategies.

Distinct pain modalities, including mechanical allodynia, thermal hyperalgesia, and spontaneous pain, may be differentially associated with specific microglial phenotypes and signaling profiles. In particular, mechanical allodynia has been closely linked to P2X4 receptor-mediated microglial activation in the spinal dorsal horn, where microglia release brain-derived neurotrophic factor (BDNF) to modulate neuronal chloride homeostasis and disinhibit nociceptive transmission (Tsuda et al., 2003). Thermal hyperalgesia, by contrast, may involve a broader neuroinflammatory milieu, with microglial production of pro-inflammatory cytokines such as IL-1β and TNF-α contributing to enhanced nociceptor sensitivity (Parusel et al., 2023). Spontaneous pain, which reflects ongoing central sensitization, has been associated with sustained microglial activation and persistent release of neuro-modulatory factors, potentially involving both pro-inflammatory and modulatory phenotypes (Inoue and Tsuda, 2018).

These results suggest that different pain symptoms may not only reflect distinct neuronal mechanisms but also correspond to heterogeneous microglial states, highlighting the importance of symptom-specific targeting of microglial signaling pathways in neuropathic pain.

## 6 Therapeutic intervention strategies targeting microglial polarization

Based on these mechanisms, restoring the balance of microglial phenotypic plasticity is increasingly viewed as a viable therapeutic approach, aiming to limit pro-inflammatory M1 activity while enhancing protective M2-associated functions.

### 6.1 Pharmacological intervention targeting key signaling pathways

#### 6.1.1 Inhibition of proinflammatory signaling pathways

Small-molecule inhibitors (e.g., the TLR4 antagonist TAK-242 and the P2X7 receptor antagonist AZD9056) can attenuate markers associated with M1-like activation and are associated with reduced pain-related behaviors in preclinical models. It is significant to recognize that microglial activation represents a dynamic and progressive continuum rather than a fixed endpoint. Therefore, most experimental manipulations are better interpreted as influencing the magnitude or qualitative features of activation states, rather than fully abolishing a distinct cellular phenotype (Kettenmann et al., 2011).

Natural compounds, including stigmasterol and β-sitosterol, have been demonstrated to attenuate neuroinflammation and pain. This effect is largely attributed to their ability to inhibit the TLR4/NF-*κ*B signaling cascade, which in turn reduces the secretion of proinflammatory cytokines (e.g., IL-1β and TNF-α) (Zheng et al., 2023; Si et al., 2024; Kang et al., 2025). Beyond its established effects at the neuromuscular junction, botulinum toxin A (BTX-A) has been reported to influence spinal microglial polarization. Specifically, inhibition of P2X7 receptor expression appears to shift the M1/M2 balance toward a more anti-inflammatory phenotype (Mao et al., 2020; Sideris-Lampretsas and Malcangio, 2022). In experimental models of neuropathic pain, BTX-A has been reported to facilitate a shift in microglial adaptive responses from a pro-inflammatory M1-like phenotype toward an anti-inflammatory M2-like phenotype. This effect appears to be associated with reduced P2X7 receptor expression and is accompanied by an elevation in pain thresholds (Mao et al., 2020). Minocycline, a prototypical broad-spectrum inhibitor of microglial activation, has demonstrated therapeutic benefits across multiple experimental pain models. Nevertheless, its limited specificity and the adverse effects associated with prolonged use underscore the need for more selective therapeutic strategies.

#### 6.1.2 Activation of protective signaling pathways

Exogenous delivery of recombinant proteins, including GDF11 (Liu and Zhang, 2025) and MFG-E8 (Zhang L. et al., 2024), as well as pharmacological activation of the κ-opioid receptor (KOR) (Liu et al., 2020) using agents such as U50488H, have been shown to facilitate the shift of microglia toward the M2-like phenotype. Mechanistically, these effects are mediated through activation of the TGF-βR1/SMAD2 and integrin β3/SOCS3 signaling cascades, or through suppression of the TLR4/NF-κB pathway. In experimental animal models, these approaches have demonstrated pronounced analgesic efficacy along with neuroprotective benefits. Both the *μ*-opioid receptor (MOR) and the κ-opioid receptor (KOR) are expressed in microglia and participate in regulating neuroinflammatory and pain-related processes. MOR signaling has been reported to facilitate pro-inflammatory microglial responses in certain contexts, thereby contributing to neuroinflammation and pain sensitization. In contrast, KOR activation is more commonly associated with reduced microglial reactivity and a shift toward anti-inflammatory signaling, which may underlie its analgesic potential. Notably, the effects of opioid receptor signaling in microglia are highly context-dependent, varying with injury type and the local microenvironment (Machelska and Celik, 2020; Cuitavi et al., 2024).

### 6.2 Novel delivery technology: application of nanocarriers

Bioactive agents (e.g., cytokines, siRNA, and miRNA) which are highly susceptible to enzymatic degradation *in vivo*, exhibit limited stability with short biological half-lives, and face considerable difficulty penetrating the blood–brain and blood–spinal cord barriers to reach spinal targets. Nanocarrier systems, including liposomes, polymer-based nanoparticles, and exosomes, can significantly improve drug delivery efficiency and prolong systemic circulation. Through surface engineering (e.g., attaching targeting peptides), they can enhance selective accumulation in the spinal cord or microglia. These strategies optimize both the precision and effectiveness of therapeutic delivery. For instance, nanoparticles encapsulating the Nrf2 gene may be administered via adipose-derived stem cells to suppress activation of the TLR4/NF-κB signaling pathway (Huang et al., 2020). Nanoparticles encapsulating miR-200c-3p have been shown to facilitate the polarization of microglia toward the M2-like phenotype within the cortical region (Yin et al., 2022). A more sophisticated strategy is to design stimulus-responsive nanocarriers. These carriers can selectively release their therapeutic payload within pain-related microenvironments, such as areas with acidic pH or high levels of reactive oxygen species. This approach allows precise spatiotemporal control over drug action (Jiang et al., 2025; Xiao et al., 2025).

### 6.3 Cell therapy and other physical interventions

#### 6.3.1 Mesenchymal stem cells (MSCs) and their exosomes

Mesenchymal stem cells (MSCs) secrete a broad array of immunomodulatory mediators (e.g., TSG-6 and PGE2) via paracrine signaling. Through these secreted factors, MSCs can drive microglia toward an M2-like phenotype and have demonstrated therapeutic benefits across multiple experimental pain models. In addition to the cells themselves, their cell-free derivatives—exosomes—serve as naturally occurring nanovesicles enriched with bioactive cargo, including proteins and miRNAs. Owing to their relatively low immunogenicity and favorable safety profile, exosomes are increasingly regarded as a promising platform for therapeutic delivery (Sideris-Lampretsas and Malcangio, 2021; Fiore et al., 2022; Hao et al., 2025).

#### 6.3.2 Physical neuromodulation

Non-invasive neuromodulatory approaches, including repetitive transcranial magnetic stimulation (rTMS) and transcranial direct current stimulation (tDCS), can directly modify cortical excitability. They also seem to affect neuroimmune activity in subcortical structures and the spinal cord. By regulating glial cell function at these levels, these techniques may help alleviate pain.

In addition to non-invasive neuromodulation, spinal cord stimulation (SCS) is an invasive but established therapy for refractory neuropathic pain. Emerging evidence suggests that SCS may exert part of its analgesic effect through modulation of spinal microglial activity. Preclinical studies have shown that SCS can reduce microglial activation markers (e.g., Iba-1, CD11b) and shift the balance toward an anti-inflammatory phenotype, although the exact mechanisms remain to be fully elucidated (Sun et al., 2022). The potential synergy between SCS and pharmacological microglial modulators represents an interesting direction for future investigation (Haight et al., 2019).

Research has indicated that cannabinoids also play a regulatory role in microglia (Duffy et al., 2021; Xu et al., 2024). Cannabinoids modulate microglial function mainly through cannabinoid receptor type 2 (CB2), which is upregulated in activated microglia during neuroinflammatory and neuropathic pain conditions. Activation of CB2 receptors generally inhibits microglial proliferation and suppresses the release of pro-inflammatory mediators such as TNF-α, IL-1β, and nitric oxide, thereby attenuating neuroinflammation. In addition, cannabinoid signaling can promote a shift toward a more anti-inflammatory or neuroprotective microglial phenotype, contributing to reduced central sensitization and pain hypersensitivity in neuropathic pain models. Endogenous cannabinoids such as 2-arachidonoylglycerol (2-AG) also participate in fine-tuning microglial responses under pathological conditions. Overall, the endocannabinoid system represents an important regulatory pathway in microglia-mediated neuroimmune interactions and pain modulation.

### 6.4 Combined strategies and multi-target interventions

Previous studies have pointed out the limitations of interventions that focus on a single target, underscoring the need for synergistic approaches capable of concurrently modulating multiple critical signaling pathways. Pain pathways are complex and overlapping, with substantial redundancy in the signaling networks involved. For this reason, combination strategies may provide greater therapeutic benefit than single-target approaches. For example, casticin can inhibit both IL-17-mediated polarization and glycolysis simultaneously (Wen et al., 2025). The combined use of MFG-E8 with integrin receptors can further enhance therapeutic efficacy (Guo et al., 2024). Combining microglia-targeted agents with conventional analgesics (e.g., gabapentin) may produce synergistic effects. Preclinical studies have demonstrated that the combination of gabapentin and minocycline produces significant synergistic analgesic effects in diabetic neuropathic pain and other experimental models of neuropathic pain (Sumracki et al., 2012; Vanelderen et al., 2013; Miranda et al., 2017). In addition, human studies suggest that minocycline, as a microglial inhibitor, can modulate neuroinflammatory states and influence pain perception. These findings indicate that a combined strategy targeting neuronal calcium channel regulation and microglial modulation may hold potential translational value for clinical pain management. A similar benefit may be achieved when these agents are paired with drugs that act on different immune pathways. Such strategies could allow dose reduction for each drug and help limit related adverse effects.

### 6.5 Current status and challenges of clinical translation

Despite the promising preclinical studies, clinical translation faces formidable challenges: (1) Target Specificity and Barrier Penetration: The key is to develop highly efficient and safe delivery systems for the CNS. These systems should precisely target microglia at lesion sites. Meanwhile, they should avoid affecting the peripheral immune system or other glial cells with essential functions in the CNS (e.g., oligodendrocytes). (2) Temporal Window and Disease Heterogeneity: Neuropathic pain (NeP) has diverse etiologies (e.g., diabetes, chemotherapy, injury) (Baron et al., 2010; Chen et al., 2022), and microglial responses may vary accordingly. The optimal timing for intervening in microglial state plasticity may vary across different etiologies and stages of pain onset. Yet the mechanisms that drive pain persistence and progression are still not fully understood. This highlights the need for more refined disease staging and the identification of specific biomarkers. (3) Model Limitations: It remains unclear whether polarization markers and functions identified in animal models (most of which use acute injury paradigms in young male rodents) (Baron et al., 2010; Chen et al., 2022) can fully represent those seen in human chronic and heterogeneous pain disorders. This question requires further validation. Studies using humanized mice, organoids, and, where feasible, analyses of post-mortem tissue or cerebrospinal fluid from patients will be vital to address this issue. (4) Safety: Whether the long-term modulation of innate immune cell function carries potential risks (e.g., increased susceptibility to infection, impaired synaptic pruning, or compromised tumor immune surveillance) requires comprehensive long-term toxicological evaluation.

Microglial activation does not follow a uniform pattern across different etiologies, and these differences likely reflect variations in the nature and duration of peripheral insults as well as the surrounding immune milieu. In diabetic peripheral neuropathy (Wang et al., 2024), chronic metabolic imbalance and oxidative stress tend to sustain a low-grade but persistent pro-inflammatory microglial state. In contrast, chemotherapy-induced neuropathy (Doyle and Salvemini, 2021) is more often associated with acute inflammatory responses and activation of pathways linked to mitochondrial dysfunction. Conditions such as postherpetic neuralgia and HIV-associated neuropathy involve virus-related immune processes (Lu et al., 2021), where microglial phenotypes appear more heterogeneous, encompassing not only classical pro-inflammatory signaling but also antiviral and immunoregulatory features. Taken together, it may be overly simplistic to view microglial activation as a single, unified response across neuropathic pain conditions. A more refined approach-integrating techniques such as single-cell sequencing and spatial transcriptomics-will be important for resolving microglial heterogeneity and its temporal dynamics, ultimately supporting the development of etiology-specific interventions and more reliable biomarkers.

At present, small-molecule antagonists targeting P2X7, TLR4 and other such targets have partially entered early-phase (Phase I/II) clinical trials for pain (Bethea and Fischer, 2021). Yet the outcomes have been inconsistent, which highlights the hurdles in predicting therapeutic efficacy in humans from animal models. Future research should validate these targets in human samples by leveraging emerging technologies, such as spatial transcriptomics and single-cell sequencing. Simultaneously, stimulus-responsive drug delivery systems need to be developed. These efforts will facilitate clinical translation in this field.

## 7 Conclusions and future perspectives

The challenge in treating neuropathic pain stems from its intricate pathological mechanisms. In Particular, the neuroimmune interactions mediated by spinal microglia are now recognized as a central framework for understanding this condition (Domoto et al., 2021; Wu et al., 2022). One of the central challenges in current research is to precisely identify the crucial functional nodes within different signaling pathways during the progression of pain. Equally important is achieving regulation that is specific in both space and time. This review offers a systematic overview of the current evidence. It shows that after peripheral nerve injury, microglia in the spinal dorsal horn are rapidly activated. Changes in the balance between pro-inflammatory M1 and anti-inflammatory M2-like phenotypes are closely linked to the development and maintenance of pain, as well as its potential resolution (Berta et al., 2016; Inoue and Tsuda, 2018). M1-polarized microglia are characterized by enhanced production of pro-inflammatory cytokines and neuroactive mediators (e.g., TNF-α, IL-1β, and BDNF), partly through activation of signaling pathways such as P2X4/P2X7 and TLR4/NF-κB. These molecular changes are closely associated with the development of central sensitization (Donnelly et al., 2020; Jiang et al., 2025; Kang et al., 2025). Conversely, microglia exhibiting M2-like features engage protective signaling cascades, including IL-10 and TGF-β/SMAD pathways, which are linked to anti-inflammatory responses and tissue repair processes (Liu F. et al., 2024). Precisely modulating this polarization balance-either by suppressing the M1-like phenotype or enhancing the M2-like phenotype (Kwok et al., 2021) has been associated with measurable reductions in pain-related behaviors in preclinical studies. This approach has identified a range of promising therapeutic targets (e.g., P2X7 and TLR4). It has also uncovered a range of intervention strategies, including small-molecule inhibitors, biologics, and nano-based delivery systems. These findings provide a foundation for developing novel analgesics (Zhao et al., 2020; Sideris-Lampretsas and Malcangio, 2022; Si et al., 2024).

Although this mechanism holds certain potential, its transformation into a clinically effective therapy is still constrained by several persistent challenges that must be addressed:

### 7.1 Beyond the M1/M2 dichotomy: toward precise characterization

Most current studies rely on the simplified M1/M2 polarization model; however, *in vivo* microglia likely display a dynamic and heterogeneous functional spectrum across different stages of pain (Paolicelli et al., 2022). Future research will need to use advanced techniques like single-cell RNA sequencing, spatial transcriptomics, and proteomics to delineate the changing molecular profile of spinal microglia and across different stages of neuropathic pain (NeP). Both human tissue samples and improved animal models will be fundamental for these analyses. Identifying distinct subpopulations with well-defined pathogenic or protective roles will facilitate the development of more precise therapeutic targets (Ma et al., 2023).

### 7.2 Decoding spatiotemporal specificity and therapeutic time windows

**Figure 2:**
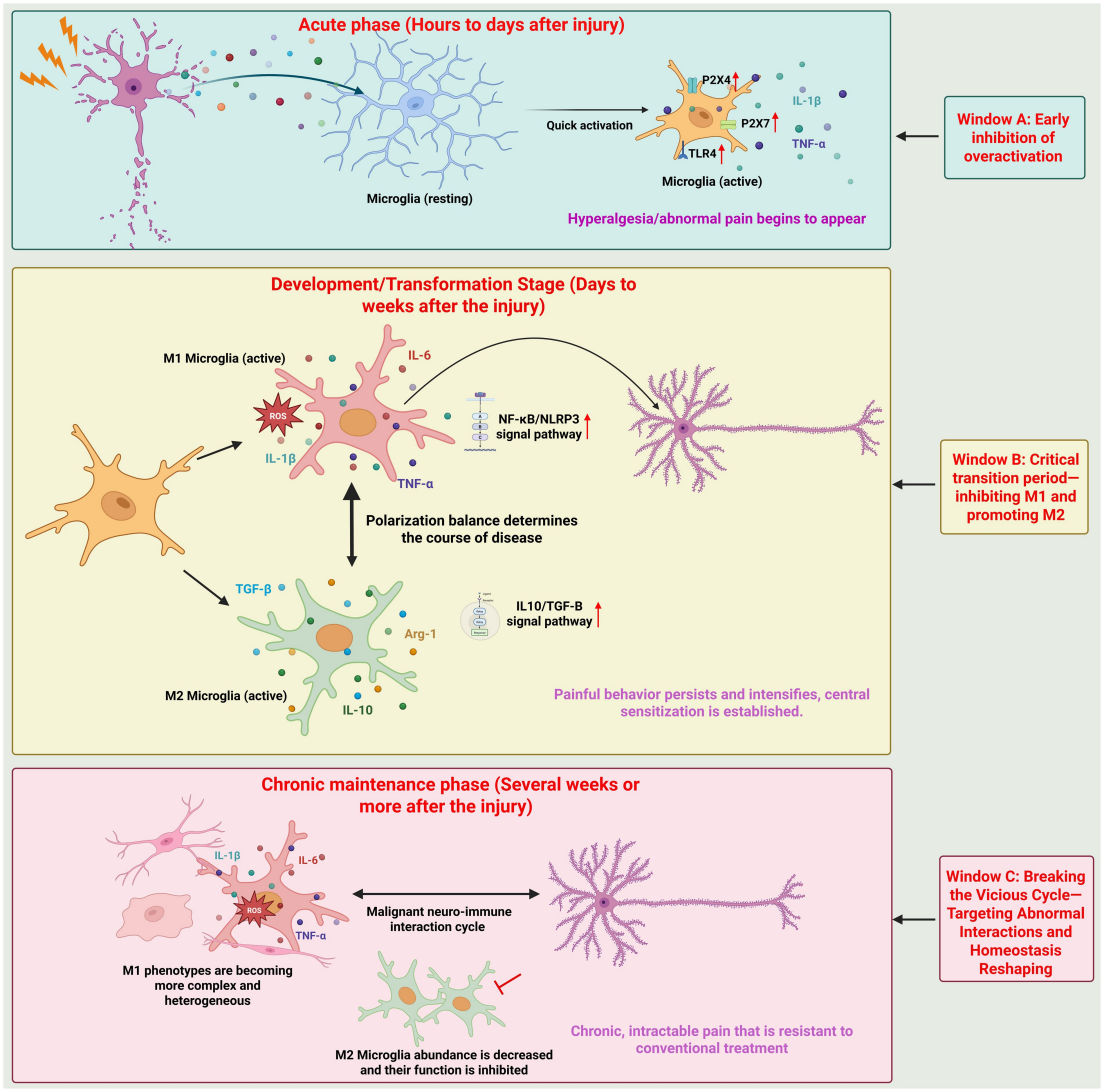
Advances in neuropathic pain and the dynamic phenotypic evolution of microglia. This schematic diagram summarizes the activation state, phenotypic polarization of spinal microglia and their dynamic relationship with pain behaviors during the process from the occurrence of nerve injury to the establishment of chronic neuropathic pain. In chronic progressive diseases, the treatment window may be wider but the effect is limited. It reveals the predominant cellular phenotypes and key molecular events at distinct phases of pain (acute phase, progression/transition phase, chronic maintenance phase), and identifies potential differential therapeutic intervention windows (e.g., early M1 inhibition, promotion of M1-to-M2 polarization, homeostatic remodeling in the chronic phase), thereby providing a conceptual framework for the development of temporally precise therapeutic strategies. Created with bioGDP.com.

The role of microglia may vary substantially across the initiation, chronification and resolution phases of pain (Ho et al., 2020) (Figure 2). Most existing interventional studies have primarily targeted preventive measures or early-phase treatment. To advance this field, it will be important to delineate the optimal “time window” for modulating microglial phenotype switching. Simultaneously, efforts should focus on creating drug delivery systems that can respond to specific pathological microenvironments. These systems would detect changes such as pH shifts or reactive oxygen species levels. By doing so, they can provide more precise control over the timing and location of therapeutic interventions (Jiang et al., 2025).

### 7.3 Incorporating sex as a biological variable

Neuroimmune regulation displays pronounced sex-specific differences, which in turn influence susceptibility to neuropathic pain (NeP), its underlying mechanisms, and responses to pharmacological treatment (Berta et al., 2016). Future basic research and clinical trials should take sex into account as an important biological factor. Studies need to clarify the shared and sex-specific mechanisms driving microglial phenotype switching. This knowledge will help lay the groundwork for more personalized analgesic strategies (see Table 1).

**Table 1:** Summary of therapeutic interventions targeting microglial polarization.

Category of intervention strategies	Target/direction of action	Representative drugs/approaches	Main mechanism of action	Research stage
Pharmacological Intervention (M1 Inhibition)	P2X7 receptor	AZD9056, A-438079	Antagonizes P2X7, inhibits NLRP3 inflammasome activation, and reduces IL-1β release	Preclinical/Phase I/II clinical
	TLR4/NF-κB pathway	TAK-242 (Resatorvid), phytosterols	Inhibits TLR4 signaling or downstream NF-κB activation, thereby reducing the expression of pro-inflammatory factors	Preclinical
	Broad-spectrum inhibitor	Minocycline	Inhibits the overall activation of microglia and the production of various pro-inflammatory mediators	Preclinical
Pharmacological Intervention (M2 Promotion)	TGF-β/SMAD pathway	GDF11 (recombinant protein)	Activates TGF-β R1, promotes SMAD2/3-mediated M2 gene expression, and inhibits NF-κB	Preclinical
	IL-10 signaling axis	Low-dose LPS (preconditioning)	Upregulates IL-10 via USP19/FOXO1 and activates the IL-10R1/STAT3 pathway	Preclinical
	κ-opioid receptor (KOR)	Dynorphin, U50488H	Activates KOR, inhibits TLR4/NF-κB signaling, and promotes M2 polarization.	Preclinical
Biologics	Integrin signaling	MFG-E8(recombinant protein)	Binds integrin β3, activates the SOCS3/STAT3 pathway, and promotes M2 polarization.	Preclinical
Novel Delivery Systems	Targeted delivery	Nanoparticles loaded with Nrf2/miRNA	Improve CNS delivery efficiency, precisely regulate the expression of specific genes (such as Nrf2), and modulate polarization.	Preclinical
	Extracellular vesicles	MSC-derived exosomes	Natural nanocarriers rich in immunomodulatory miRNAs and proteins, inducing the M2 phenotype.	Preclinical
Combined/Multi-Target Strategies	Multi-pathway synergy	Casticin	Simultaneously inhibits IL-17 signaling and glycolysis (PKM2/STAT3), enabling multi-dimensional regulation.	Preclinical
	Synergistic analgesia	MFG-E8 and Integrin Agonist	Combined medication to enhance the induction effect on M2 polarization.	Preclinical
Non-Pharmacological Intervention	Physical Regulation	Repetitive Transcranial Magnetic Stimulation (rTMS)	Modulates cortical excitability and may indirectly affect the activity of subcortical and spinal glial cells.	Clinical application/Mechanistic research

This table summarizes current intervention strategies targeting the polarization balance of microglia in neuropathic pain, including their targets, representative agents/methods, primary mechanisms, and research stages.

### 7.4 Bridging the translational gap from preclinical to clinical settings

Although preclinical findings have been encouraging, moving from experimental validation to patient care still involves considerable practical and regulatory hurdles. Major challenges include the limited ability of drugs or formulations to cross the blood–brain and blood–spinal cord barriers. There is also the risk of unforeseen effects on other CNS cell types and potential safety concerns from long-term immune modulation. Moreover, animal model behaviors often do not fully capture the subjective experience of pain in humans (Deng et al., 2025). Strengthening interdisciplinary collaboration, adopting more predictive translational models such as organoids and humanized mouse models, and initiating biomarker-guided exploratory clinical trials at an early stage may represent effective strategies for overcoming current translational barriers.

Understanding the neuroimmune processes linked to microglial state specification may help explain the dysregulated inflammatory responses underlying neuropathic pain. Further progress in this field requires moving beyond the traditional dichotomous framework toward a more dynamic spectrum. It also depends on methodological improvements, including multi-omics integration and more precise delivery strategies. Furthermore, study designs should better address sex differences and enhance translational relevance. A more integrated understanding of neuroimmune regulation may facilitate the development of pain therapies that better modulate immune–neural interactions and improve symptom control in chronic pain conditions.
