# Supplementary material for: The mechanism of dynamic switching between M1/M2 phenotypes of microglia in neuropathic pain: a narrative review
Source: Front Mol Neurosci. 2026 May 26;19:1834555. doi: 10.3389/fnmol.2026.1834555 (PMC13246711; doi:10.3389/fnmol.2026.1834555)

Supplementary Material

Detailed Literature Search Strategy

This supplementary material provides the complete search strategy employed for the review entitled “The Mechanism of Dynamic Switching Between M1/M2 Phenotypes of Microglia in Neuropathic Pain: A Narrative Review” (Manuscript ID: 1834555).

Table S1. Summary of Search Strategy

**Parameter**	**Details**
**Databases**	PubMed, Web of Science Core Collection, Scopus
**Time frame**	Database inception to December 31, 2025
**Language**	English
**Publication types**	Original research articles, review articles, systematic reviews, clinical trials, case series (≥5 cases)
**Excluded publication types**	Conference abstracts, editorials, commentaries, letters, opinion pieces, book chapters
**Species**	Human and animal (rodent, non-human primate) studies
**Search fields**	Title, Abstract, Keywords (or equivalent: MeSH, EMTREE, Topic)

Table S2. Inclusion and Exclusion Criteria

**Criterion**	**Inclusion**	**Exclusion**
**Topic focus**	Microglial polarization in neuropathic pain (central or peripheral origin)	Microglial polarization in other CNS disorders without pain focus (e.g., Alzheimer's disease, stroke, traumatic brain injury)

Criterion	Inclusion	Exclusion
**Mechanistic relevance**	Studies addressing signaling pathways (P2X7, TLR4/NF-κB, cGAS-STING, IL-10/TGF-β, metabolic regulation) that directly regulate M1/M2-like transitions in the context of pain	Studies on microglial chemotaxis, proliferation, or survival without explicit polarization analysis
**Intervention focus**	Pharmacological, biological, nanocarrier, cell-based, or neuromodulatory interventions targeting microglial polarization for pain relief	Purely behavioral pain studies without cellular/molecular readouts; studies on non-microglial cells (astrocytes, neurons, peripheral macrophages) without parallel microglial analysis
**Study design**	Original research (in vivo, ex vivo, in vitro), systematic reviews, high-quality narrative reviews	Conference abstracts, editorials, opinion pieces, case reports (n<5)
**Species**	Human, rodent, non-human primate	Other species (zebrafish, drosophila, C. elegans) without validated pain-related behavioral correlates
**Publication status**	Peer-reviewed, published, in-press (accepted manuscripts)	Preprints, non-English publications, duplicate publications

Figure S1. PRISMA-Style Flow Diagram of Study Selection

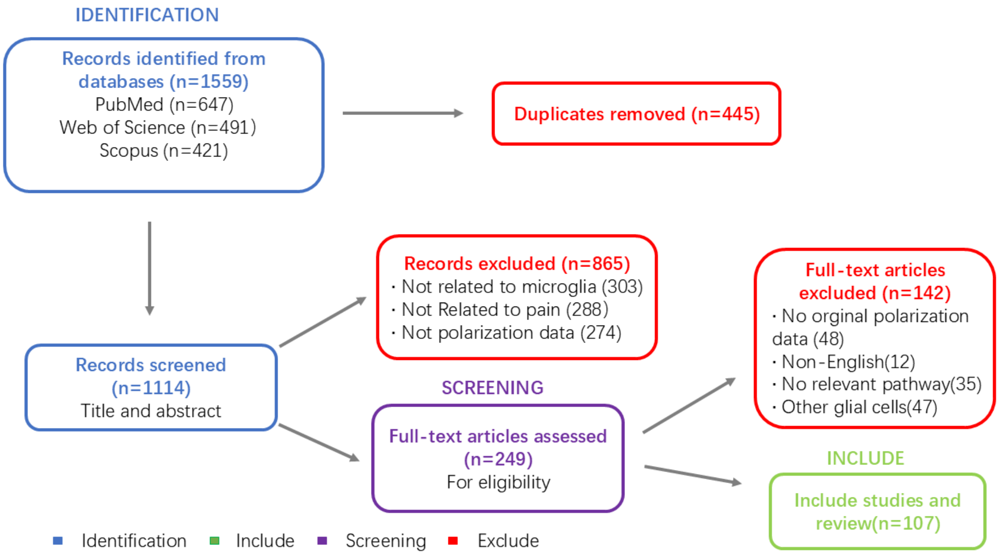

Supplement: Supplementary file 1 [file Data_Sheet_1.PDF]
